# Predictions for the ${\varvec{\Lambda }_b \rightarrow J/\psi ~ \Lambda (1405)}$ decay

**DOI:** 10.1140/epjc/s10052-015-3438-1

**Published:** 2015-05-19

**Authors:** L. Roca, M. Mai, E. Oset, Ulf-G. Meißner

**Affiliations:** Departamento de Física, Universidad de Murcia, 30100 Murcia, Spain; Helmholtz-Institut für Strahlen- und Kernphysik (Theorie), Universität Bonn and Bethe Center for Theoretical Physics, 53115 Bonn, Germany; Departamento de Física Teórica and IFIC, Centro Mixto Universidad de Valencia-CSIC, Institutos de Investigación de Paterna, Aptdo. 22085, 46071 Valencia, Spain; Forschungszentrum Jülich, Institut für Kernphysik, Institute for Advanced Simulation, Jülich Center for Hadron Physics, 52425 Jülich, Germany

## Abstract

We calculate the shape of the $\pi \Sigma$ and $\bar{K} N$ invariant mass distributions in the $\Lambda _b \rightarrow J/\psi \, \pi \Sigma$ and $\Lambda _b \rightarrow J/\psi \,\bar{K} N$ decays that are dominated by the $\Lambda (1405)$ resonance. The weak interaction part is the same for both processes and the hadronization into the different meson–baryon channels in the final state is given by symmetry arguments. The most important feature is the implementation of the meson–baryon final-state interaction using two chiral unitary models from different theoretical groups. Both approaches give a good description of antikaon–nucleon scattering data, the complex energy shift in kaonic hydrogen and the line shapes of $\pi \Sigma K$ in photoproduction, based on the two-pole scenario for the $\Lambda (1405)$. We find that this reaction reflects more the higher mass pole and we make predictions of the line shapes and relative strength of the meson–baryon distributions in the final state.

## Introduction

The nonleptonic weak decays of charmed and bottom hadrons are turning into a useful tool to learn about the nature of hadrons. Although weak interactions violate parity and isospin, the dominance of certain mechanisms at the quark level induced by the topology of the mechanisms and the strength of the different Cabibbo–Kobayashi–Maskawa (CKM) matrix elements, allows one to select certain decays modes that turn out to be sensitive to the production of some particular hadrons; see e.g. Refs. [1–3]. In this way, surprises are found like the strong signal of the $f_0(980)$ in $B^0_s$ decay into $J/\psi$ and $\pi ^+ \pi ^-$ [4, 5], while no signal was found for the $f_0(500)$. This is surprising, since the $f_0(500)$ couples more strongly to $\pi ^+ \pi ^-$ than the $f_0(980)$. Further, in the decay of $\bar{B}^0$ into $J/\psi$ and $\pi ^+ \pi ^-$ [6], the $f_0(500)$ signal was prominent while the $f_0(980)$ production was strongly suppressed. Attempts to explain these features in terms of tetraquark structures for the scalar mesons were made in [7]. A different line of investigation has been opened in [8] following the findings of chiral unitary theory, where these scalar mesons are dynamically generated from the interaction of pseudoscalar mesons [9–14]. In this approach, the basic mechanism at the quark level is identified as follows: one $c \bar{c}$ state forms the $J/\psi$, another $q \bar{q}$ pair hadronizes into a pair of mesons, and the final-state interaction of these mesons leads to the production of the scalar resonances. It should be mentioned that the use of unitarized chiral perturbation theory (ChPT) to explore the physics of heavy meson decays was pioneered in Refs. [15, 16], and this has recently been employed to quantify the S-wave pollution in semileptonic B decays [17] and to facilitate the extraction of $|V_{ub}|$ from $B_{\ell 4}$ decays [18].

The method of Ref. [8] has allowed one to interpret many other different decays. In this sense, ratios for the production of $J/\psi$ and vector mesons in $B$ decays were evaluated in [19] and predictions for the $J/\psi \kappa (800)$ decay were also made. In [20] the $D^0$ decays into $K^0_s$ and $f_0(500)$, $f_0(980)$, $a_0(980)$ were described. Dynamically generated states from the vector–vector interaction were investigated in the $\bar{B}^0$ and $\bar{B}^0_s$ decays into $J/\psi$ plus $f_0(1370),~f_0(1710),~f_2(1270),~f'_2(1525),~K^*_2(1430)$ [21]. Similarly, the $\bar{B}^0$ decay into $D^0$ and $\rho$ or $f_0(500)$, $f_0(980)$, $a_0(980)$ and $\bar{B}^0_s$ decays into $D^0$ and $K^{*0}$ or $\kappa (800)$ were addressed in [22]. Further work was done in [23], where the $KD$ scattering and the $D_{s0}^*(2317)$ resonance were studied from the $B^0_s$ decay into $D_s~ DK$. Also, semileptonic $B_s$ and $B$ decays were addressed in [24].

In the present work we would like to follow this same line of reasoning but involving baryons rather than mesons. The reaction we study here is $\Lambda _b \rightarrow J/\psi ~ \Lambda (1405)$, where the $\Lambda (1405)$ is to be seen in the $\pi \Sigma$ spectrum. This reaction is not measured yet but the related process $\Lambda _b \rightarrow J/\psi ~ \Lambda (1115)$ has already been measured by the D0 [25] and the ATLAS [26] collaborations. Further, there is experimental information on the $\Lambda _b \rightarrow J/\psi ~ K^- p$ decay channel from the LHCb [27, 28] and CDF [29] collaborations. No absolute values are provided for the latter decay and only ratios to other reactions are studied. Our work will allow us to relate the $\Lambda _b \rightarrow J/\psi ~ \Lambda (1405)$ decay to the $\Lambda _b \rightarrow J/\psi ~ K^- p$ decay and ratios between the invariant $K^- p$ and $\pi \Sigma$ mass distributions will be provided.

The reason to suggest the measurement of the $\Lambda (1405)$ in the $\Lambda _b$ decay is the relevance of the $\Lambda (1405)$ as the most significant example of a dynamically generated resonance. Indeed, very early it was already suggested that this resonance should be a molecular state of $\bar{K} N$ and $\pi \Sigma$ [30, 31]. This view has also been invoked in Ref. [32]. However, it was with the advent of chiral unitary theory that this idea gained strength [33–47].

One of the surprises of these works is that two poles were found for the $\Lambda (1405)$.^1^ The existence of two states was hinted in [48], using the chiral quark model, and it was found in [36] using the chiral unitary approach. A thorough search was conducted in [40] by looking at the breaking of SU(3) in a gradual way, confirming the existence of these two poles and its dynamical origin. One of the consequences of this two-pole structure is that the peak of the resonance does not always appear at the same energy, but varies between 1420 and 1480 MeV depending on the reaction used [49–56]. This is because different reactions give different weights to each of the poles. While originally most reactions gave energies around 1400 MeV, the origin of the nominal mass of the resonance, the $K^- p \rightarrow \pi ^0\pi ^0 \Sigma ^0$ was measured [52] and a peak was observed around 1420 MeV, narrower than the one observed in [49, 50], which was interpreted within the chiral unitary approach in [57]. Another illustrative experiment was the one of [58] where a clear peak was observed around 1420 MeV in the $K^- d \rightarrow n \pi \Sigma$ reaction, which was also interpreted theoretically in [59] along the same lines; see also Refs. [60, 61]. Very recently it has also been suggested that the neutrino induced production of the $\Lambda (1405)$ is a good tool to further investigate the properties and nature of this resonance [62].

The basic feature in the dynamical generation of the $\Lambda (1405)$ in the chiral unitary approach is the coupled-channel unitary treatment of the interaction between the coupled channels $K^-p$, $\bar{K}^0n$, $\pi ^0\Lambda$, $\pi ^0\Sigma ^0$, $\eta \Lambda$, $\eta \Sigma ^0$, $\pi ^+\Sigma ^-$, $\pi ^-\Sigma ^+$, $K^+\Xi ^-$ and $K^0\Xi ^0$. The coupled-channels study allows us to relate the $K^-p$ and $\pi \Sigma$ production, where the resonance is seen, and this is a unique feature of the nature of this resonance as a dynamically generated state. It allows us to make predictions for the $\Lambda (1405)$ production from the measured $\Lambda _b \rightarrow J/\psi ~ K^-p$ decay.

Technically, the work proceeds as follows: the basic mechanism for the $\Lambda _b \rightarrow J/\psi ~ K^- p$ decay at the quark level is identified. First, a $c \bar{c}$ state is produced, which forms the $J/\psi$, and the three remaining light quarks $u,d,s$ hadronize to a meson–baryon pair. After this, the latter undergoes final-state interactions in coupled channels, such that the $\Lambda (1405)$ is unavoidably produced. To calculate the corresponding decays, we shall use two different models of the coupled-channels interaction: One of them [63, 64] uses the lowest order chiral Lagrangians slightly modified to fit the photoproduction data from CLAS [53, 54]. The other one incorporates explicitly the next-to-leading order Lagrangian with coefficients that are also fitted to the same data [65]. The latter approach has been used to generate theoretical uncertainties, which are important for judging the precision achieved. In spite of the apparent differences, the results for different observables are remarkably similar in both approaches and the two poles obtained are practically identical and quite similar to those obtained in [40].

This is the first theoretical work done for this reaction, yet it shares some aspects with a similar process, the $\Lambda _c \rightarrow \pi ^+ \pi ^- \Sigma$ reaction, which was proposed in [66] as a tool to measure the $\pi ^- \Sigma$ scattering length. Indeed, in [66] the hadronization of the final three quark state at the tree level was done, albeit in a different way, and the final-state interaction of coupled channels is described in a similar manner as done here. Other works for related reactions used quark models to evaluate amplitudes, like in the study of the $\Lambda _b \rightarrow J/\psi ~ \Lambda (1115)$ reaction [67], or the semileptonic transitions from $\bar{B}_s \rightarrow K l \bar{\nu }_l$ [68]. Further, some studies made use of heavy quark effective theory to evaluate related amplitudes as for the process $\Lambda _b \rightarrow \Lambda _c l \bar{\nu }_l$ [69]. In contrast to these later works, the one presented here, as well as the one of [66], does not perform a microscopic study of the reaction, since we do not aim at obtaining absolute rates; instead we exploit the dynamics of the coupled channels to relate the distributions of invariant masses in different final states, hopefully contributing to a better understanding of the meson–baryon interaction and the nature of some resonances, in particular the $\Lambda (1405)$.

## Formalism

In this section we describe the reaction mechanism for the process $\Lambda _b \rightarrow J/\psi \,\Lambda (1405)$, which is divided into three parts. The first two parts describe the decay mechanism $\Lambda _b\rightarrow J/\psi \,B\phi$, with $B\phi$ the meson–baryon system of strangeness $S=-1$, in the language of the quark model. Then, after hadronization, the final-state interaction is described in terms of the effective (hadronic) degrees of freedom of ChPT. After a resummation of the chiral meson–baryon potential to an infinite order, the $\Lambda (1405)$ is generated dynamically. In the following, we describe each single step of this reaction mechanism in more detail.

**Fig. 1:**
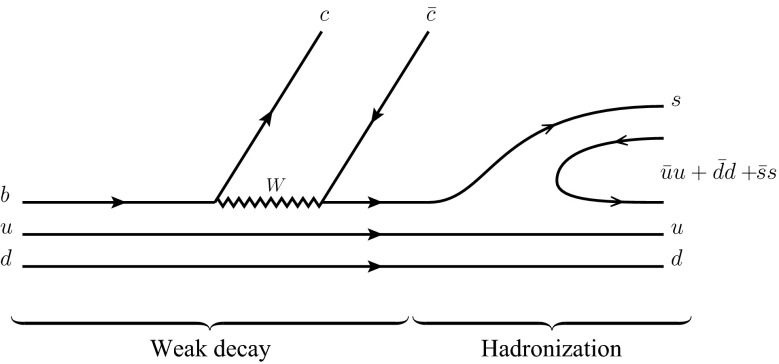
Production of a $K^-p$ pair from the weak decay ${\Lambda _b\rightarrow \Lambda \,J/\psi }$ via a hadronization mechanism. The *full* and *wiggly lines* correspond to quarks and the $W$-boson, respectively

*Weak decay* The $b$ quark of the $\Lambda _b$ undergoes the weak transition to a $c\bar{c}$ pair and an $s$-quark as depicted in the left part of Fig. 1. This transition is quantified by the matrix elements of the CKM matrix $V_{cb}V_{cs}^*$ and it is favored compared to $b\rightarrow c\bar{c} d$ leading to the $\Lambda _b\rightarrow J/\psi p\pi ^-$, which was observed for the first time by the LHCb collaboration; see Ref. [27].

*Hadronization* The $$c \bar{c}$$ pair forms the well-known $$J/\psi $$, while the virtual $$uds$$ three-quark state undergoes hadronization to form a meson–baryon pair. This happens due to the large phase space available ($$\le $$$$2522$$ MeV for $$M_{\Lambda _b}=5619$$ MeV, $$M_{J/\psi }=3097$$ MeV), so that a quark–antiquark pair can become real, forming together with the three available quarks a meson–baryon pair. In principle, different meson–baryon states can be produced in such a mechanism. To determine their relative significance, we assume first that the $$u$$ and $$d$$ quarks of the original $$\Lambda _b$$ state are moving independently in a potential well. Further, we note that the $$\Lambda _b$$ ($$J^p=1/2^+$$) is in the ground state of the three-quarks $$(udb)$$. Therefore, all relative angular momenta between different quarks are zero. After the weak transition, but before the hadronization, the three-quark state $$(uds)$$ has to be in a p-wave since the final $$\Lambda (1405)$$ is a negative-parity state. On the other hand, since the $$u$$ and $$d$$ quarks are considered to be spectators and they were originally in $$L=0$$, the only possibility is that the $$s$$ quark carries the angular momentum, $$L=1$$. Moreover, since the final mesons and baryons are in the ground state and in s-wave to each other, all the angular momenta in the final state are zero. Consequently, the $$\bar{q} q$$ pair cannot be produced elsewhere, but between the $$s$$ quark and the $$ud$$ pair as depicted in Fig. 1.

The flavor state of the initial $\Lambda _b$ can be written as

$$\begin{aligned} |\Lambda _b\rangle =\frac{1}{\sqrt{2}}|b(ud-du)\rangle , \end{aligned}$$

turning after the weak process into

$$\begin{aligned} \frac{1}{\sqrt{2}}|s(ud-du)\rangle , \end{aligned}$$

since the $u$ and $d$ quarks are considered to be spectators. Thus, after hadronization, the final quark flavor state is

$$\begin{aligned} |H\rangle&\equiv \frac{1}{\sqrt{2}}|s\,(\bar{u} u +\bar{d} d +\bar{s} s)\,(ud-du)\rangle \\&=\frac{1}{\sqrt{2}} \sum _{i=1}^3{|P_{3i}q_i(ud-du)}\rangle , \end{aligned}$$

where we have defined

$$\begin{aligned} q\equiv \left( \begin{array}{c}u\\ d\\ s\end{array}\right) \quad \text {and}\quad P\equiv q\bar{q}^\tau =\left( \begin{array}{ccc}u\bar{u} &{} u\bar{d} &{} u\bar{s}\\ d\bar{u} &{} d\bar{d} &{} d\bar{s}\\ s\bar{u} &{} s\bar{d} &{} s\bar{s} \end{array}\right) . \end{aligned}$$

The latter is nothing else than the quark–antiquark representation of the $\mathrm{SU}(3)$ pseudoscalar meson matrix

$$\begin{aligned} P= \left( \begin{array}{ccc} \frac{\pi ^0}{\sqrt{2}} + \frac{\eta }{\sqrt{3}}+\frac{\eta '}{\sqrt{6}}&{} \pi ^+ &{} K^+\\ \pi ^-&{} -\frac{1}{\sqrt{2}}\pi ^0 + \frac{\eta }{\sqrt{3}}+ \frac{\eta '}{\sqrt{6}}&{} K^0\\ K^-&{} \bar{K}^0 &{} -\frac{\eta }{\sqrt{3}}+ \frac{2\eta '}{\sqrt{6}} \end{array} \right) , \end{aligned}$$

where we have assumed the ordinary mixing between the singlet and octet $\mathrm{SU}(3)$ states for the $\eta$ and $\eta '$; see e.g. Ref. [70]:

$$\begin{aligned} \eta = \frac{1}{3}\eta _1 + \frac{2\sqrt{2}}{3}\eta _8, \quad \eta ' = \frac{2\sqrt{2}}{3}\eta _1 - \frac{1}{3}\eta _8. \end{aligned}$$

The hadronized state $|H\rangle$ can now be written as

$$\begin{aligned} |H\rangle&= \frac{1}{\sqrt{2}} ( K^- u(ud-du)+\bar{K}^0 d(ud-du)\\&\quad +\frac{1}{\sqrt{3}}(-\eta +\sqrt{2}\eta ')s(ud-du)). \end{aligned}$$

We can see that these states have overlap with the mixed antisymmetric baryon state [71]. Further, the flavor states of the final octet baryons can be written as

$$\begin{aligned} |p \rangle&=\frac{1}{\sqrt{2}}|u(ud-du)\rangle ,\\ |n \rangle&=\frac{1}{\sqrt{2}}|d(ud-du)\rangle ,\\ |\Lambda \rangle&=\frac{1}{\sqrt{12}}| (usd-dsu)+(dus-uds) +2(sud-sdu)\rangle . \end{aligned}$$

Consequently, the hadronized state can be expressed in terms of ground state octet mesons and baryons as

1 $$\begin{aligned} |H\rangle =|K^-p\rangle +|\bar{K}^0 n\rangle -\frac{\sqrt{2}}{3}|\eta \Lambda \rangle +\frac{2}{3}|\eta '\Lambda \rangle , \end{aligned}$$

which provides the relative weights between the final meson–baryon channels. Note that there is not direct production of $\pi \Sigma$ and $K\Xi$, however, these channels are present through the intermediate loops in the final-state interaction as described below. Moreover, the final $\eta '\Lambda$ channel will be neglected since it has a small effect due its high mass and can be effectively reabsorbed in the regularization parameters as will be explained below.

**Fig. 2:**
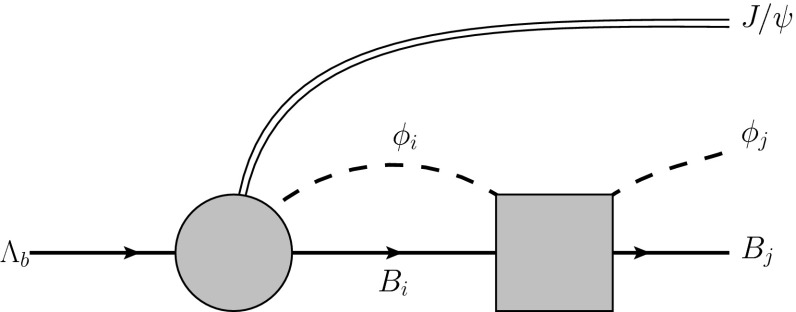
Final-state interaction of the meson–baryon pair, where the *double*, *full* and *dashed lines* denote the $J/\psi$, the baryons and the pseudoscalar mesons, respectively. The *circle* and *square* denote the production mechanism of the $J/\psi B_i\phi _i$ as depicted in Fig. 1 as well as meson–baryon scattering matrix $t_{ij}$, respectively

*Formation of the*${\varvec{\Lambda }(1405)}$ After the production of a meson–baryon pair, the final-state interaction takes place, which is parametrized by the scattering matrix $t_{ij}$, see Fig. 2. Thus, after absorbing the CKM matrix elements and kinematic prefactors into an overall factor $V_p$, the amplitude $\mathcal {M}_{j}$ for the transition $\Lambda _b\rightarrow J/\psi \,\phi _jB_j$ can be written as

2 $$\begin{aligned} \mathcal {M}_{j}(M_\mathrm{inv})=V_p\left( h_j+\sum _{i}h_iG_i(M_\mathrm{inv})\,t_{ij}(M_\mathrm{inv}) \right) , \end{aligned}$$

where, considering Eq. (1),

$$\begin{aligned} h_{\pi ^0\Sigma ^0}&=h_{\pi ^+\Sigma ^-}=h_{\pi ^-\Sigma ^+}=0,\quad h_{\eta \Lambda }= -\frac{\sqrt{2}}{3},\\ h_{K^-p}&=h_{\bar{K}^0n}=1, \quad h_{K^+\Xi ^-}=h_{K^0\Xi ^0}=0, \end{aligned}$$

and $G_i$ denotes the one-meson–one-baryon loop function, chosen in accordance with the models for the scattering matrix^2^$t_{ij}$ as will be described below. Further, $M_\mathrm{inv}$ is the invariant mass of the meson–baryon system in the final state. Note also that the above amplitude holds for an s-wave only and every intermediate particle is put on the corresponding mass shell. Finally, the invariant mass distribution $\Lambda _b\rightarrow J/\psi \,\phi _jB_j$ reads

3 $$\begin{aligned} \frac{\mathrm{d}\Gamma _j}{\mathrm{d}M_\mathrm{inv}}(M_\mathrm{inv}) =\frac{1}{(2\pi )^3}\frac{m_j}{M_{\Lambda _b}}{\mathbf {p}}_{J/\psi } {\mathbf {p}}_j\left| \mathcal {M}_{j}(M_\mathrm{inv})\right| ^2, \end{aligned}$$

where $\mathbf{p }_{J/\psi }$ and $\mathbf{p }_j$ denote the modulus of the three-momentum of the $J/\psi$ in the $\Lambda _b$ rest-frame and the modulus of the center-of-mass three-momentum in the final meson–baryon system, respectively. The mass of the final baryon is denoted by $m_j$.

The factor $$V_p$$ in Eq. (2) takes into account the weak interaction process, containing matrix elements of the two standard $$\gamma ^\mu (1-\gamma _5)$$$$Wqq$$ vertices and the $$W$$ propagator [72]. In addition it also contains matrix elements for the hadronization process. However, we only use the flavor structure of the latter process and the remaining dynamical factors are included in $$V_p$$, which is then taken constant, independent on $$M_\mathrm{inv}$$. Actually $$V_p$$ contains form factors that depend on the momentum transfer, or $$M_\mathrm{inv}$$ equivalently, but evaluations of this form factor in the related reactions $$B^0_s\rightarrow J/\psi f_0(500) (f_0(980))$$ indicate that it is very weakly dependent on $$M_\mathrm{inv}$$ over a wide range of energies from $$M_\mathrm{inv}=$$ 500–1000 MeV [73]. In addition, in Ref. [74] it was found from an analysis of the experiment that the ratio of form factors $$F^{f_0(980)}_{B^0_s}(m^2_{J/\psi })/F^{f_0(500)}_{B^0}(m^2_{J/\psi })$$ is compatible with unity. In our case the range of $$M_\mathrm{inv}$$ where we are interested is about 100 MeV and this justifies to take $$V_p$$ constant.

As already described in the introduction, the baryonic $J^P=1/2^-$ resonance $\Lambda (1405)$ has to be understood as a dynamically generated state from the coupled-channel effects. The modern approach for it is referred to as chiral unitary models; see e.g. Refs. [33–47]. In the present approach we use the scattering amplitude from two very recent versions of such approaches; see Refs. [63–65]. While the basic motivation is the same for both approaches, there are several important differences, which shall be described in the following two subsections.

### Summary of the Bonn model

The model described in the present subsection has been developed originally in Ref. [75] and used first for the analysis of the lowest $S_{11}$ nucleon resonances from scattering data as well as single-meson photoproduction data in Ref. [76]. Later in Ref. [47] it was also applied to meson–baryon scattering in the strangeness $S=-1$ sector, adjusting the free parameters of the model to the available scattering data (including the threshold data from kaonic hydrogen). While the data was described quite satisfactorily, the broad pole of the $\Lambda (1405)$ appeared at a different position than usually assumed. While the reason for this discrepancy may have various roots, see the discussion in Ref. [47], one important systematic observation was made there. Namely, the off-shell contributions of the intermediate particle fields in the Feynman diagrams are quite moderate in this setting. This observation is enormously useful as it allows one to reduce the computational effort by a factor of 30–60 and therefore to study the large parameters space of this model in more detail as was done in Ref. [65]. There, in a large scale analysis of the parameter space we have found several solutions including similar ones to that of Ref. [47]. However, in a very conservative test against the recent and very precise two-meson photoproduction data by the CLAS collaboration [53, 54] many solutions were ruled out. The best solution of this procedure is used here. In what follows we will describe the major features of this approach, while for details the reader is referred to Refs. [47, 65, 75].

The driving term of this model is the chiral potential, derived from the leading and next-to-leading order chiral Lagrangian in the three flavor formulation; see Ref. [77]. In the on-shell approximation, this potential reads

4 $$\begin{aligned} V({/\!\!{p}})=A(p^2)+B(p^2){/\!\!{p}}~, \end{aligned}$$

with

$$\begin{aligned} A(p^2)&=\Big (-A_{WT}(m_{i}+m_{f})+A_{14}(q_{i}\cdot q_{f})\\&\quad +2A_{57}\big ((q_{i}\cdot q_{f})-p^2-m_{i}m_{f}\big )\\&\quad -A_{811}\big (m_{f}(q_{i}\cdot p)+m_{i}(p\cdot q_{f})\big )+A_M \Big ),\\ B(p^2)&=\Big (2A_{WT}\\&\quad +2A_{57}(m_{i}+m_{f})+A_{811}\big ((q_{i}\cdot p)+(p\cdot q_{f})\big )\Big ), \end{aligned}$$

where here and in the following $M/m$ and $q/p$ denote the meson/baryon mass and the meson/overall four-momentum, respectively, with $p^2 = M_\mathrm{inv}^2$. The index $i/f$ denotes the in-/out-going states. The $A_{WT}$, $A_{14}$, $A_{57}$, $A_{M}$ and $A_{811}$ are ten-dimensional matrices which encode the coupling strengths between all ten channels of the meson–baryon system for strangeness $S=-1$, i.e. $\{K^-p$, $\bar{K}^0 n$, $\pi ^0\Lambda$, $\pi ^0\Sigma ^0$, $\pi ^+\Sigma ^-$, $\pi ^-\Sigma ^+$, $\eta \Lambda$, $\eta \Sigma ^0$, $K^+\Xi ^-$, $K^0\Xi ^0\}$. They are given explicitly in Ref. [65]. Setting all meson masses and decay constants to their physical values, the only unknown of the above equation are the 14 low-energy constants (LECs) of SU(3) ChPT at NLO. These LECs serve as free parameters of the present model as they are not known precisely at the moment.

At any finite order, the strict chiral expansion of the scattering amplitude in the baryon sector is restricted to a certain range around the point $p^2=m_0^2$ (with $m_0$ the octet mass in the chiral limit) and a small momentum transfer to the baryon. Moreover, at any finite order such a series fails in the vicinity of resonances such as the $\Lambda (1405)$, located just below the $\bar{K} N$ threshold. Therefore, a resummation of the driving term is required to describe this system. In the present work we use the coupled-channel Bethe–Salpeter equation in the on-shell approximation. Here, the scattering amplitude $T({/\!\!{p}})$ is the solution of the following matrix equation over the $10$-dimensional channel space:

5 $$\begin{aligned} T({/\!\!{p}})=V({/\!\!{p}})+V({/\!\!{p}})\,G(M_\mathrm{inv})\,T({/\!\!{p}}), \end{aligned}$$

where $G$ is a diagonal matrix, containing the one-meson-one-baryon loop functions as elements, which on-shell read

6 $$\begin{aligned} G^{ij}(M_\mathrm{inv})=i\int \frac{d^dl}{(2\pi )^d}\frac{2m_i\delta ^{ij}}{(l^2-M_i^2+i\epsilon )((l-p)^2-m_i^2+i\epsilon )}. \end{aligned}$$

This function is treated in dimensional regularization, applying the usual $\overline{\mathrm{MS}}$ subtraction scheme. It should be noted that due to the non-perturbative character of Eq. (5) the regularization scale is treated as a free parameter of the model. In the isospin basis, there are six such parameters. All free parameters of the model are taken from the solution #4 from Ref. [65], which was found to be the best solution, describing all available meson–baryon scattering data as well as the recent two-meson photoproduction data by the CLAS collaboration [53, 54]. For the purpose of the present work, the scattering amplitude $T({/\!\!{p}}\,)$ of this solution is projected to the lowest partial wave, i.e. $f_{0+}$. The latter is related to the scattering matrix $t_{ij}$ from Eq. (2) via

7 $$\begin{aligned} t^{ij}(M_\mathrm{inv})=-\frac{4\pi M_\mathrm{inv}}{\sqrt{m_i m_j}}f_{0+}^{ij}(M_\mathrm{inv}). \end{aligned}$$

For completeness, we recall that two poles of $\Lambda (1405)$ were found for this solution, located on the second Riemann sheet connected to the first one between the $\pi \Sigma$ and $\bar{K}N$ thresholds. Their positions are $({1429^{+8}_{-7}-i\,12^{+2}_{-3}})$ MeV and $({1325^{+15}_{-15}-i\,90^{+12}_{-18}})$ MeV. Here, the error bars are due to fit parameter errors. Naturally, the latter lead to an uncertainty of the scattering amplitude $t^{ij}(M_\mathrm{inv})$, which is discussed in detail in Ref. [65]. The focus of the present work lies on the systematic error, considering two different models for the final-state interactions in the $\Lambda _b$ decay, and we will omit these parameter errors in what follows.

### Summary of the MV model

Let us briefly review the second unitarized meson–baryon model [63, 64] that we are going to use in the present work (which we will call MV model, after Murcia–Valencia, in the following), for the sake of completeness and to ease the comparison with the Bonn model summarized in Sect. 2.1. The aim of the studies carried out in Refs. [63, 64] was to fine tune the meson–baryon scattering amplitudes obtained in the chiral unitary approach by allowing to change slightly the unitarization kernel and loop functions through the inclusion of free parameters of natural order which were fitted to the $\gamma p \rightarrow K^+ \pi \Sigma$ data from CLAS.

The basic model for the unitarized meson–baryon scattering amplitude has been widely developed and applied in many previous works (see for instance [35, 36, 38, 78]). The chiral unitary approach is based on the implementation of unitarity and the exploitation of the analytic properties of the scattering amplitudes with the only input of the lowest orders chiral potentials. This has been usually carried out by means of the inverse amplitude method [10, 79] or the N/D method [36, 80, 81] which was shown in [9] to be equivalent to the Bethe–Salpeter equation. From the $N/D$ method, the scattering amplitude $t_{ij}$ fulfills Eq. (5) which provides the solution

8 $$\begin{aligned} t=[1-vG]^{-1}v, \end{aligned}$$

in the normalization of Eq. (7), with $v_{ij}$ the $s$-wave projected meson–baryon potential described below, Eq. (9).

In the MV model, the interaction kernel $v_{ij}$ obtained from the lowest order chiral Lagrangian for the interaction of the octet of pseudoscalar mesons with the octet of the lowest mass $1/2^+$ baryons [82]. The $s$-wave projected potential reads [38]

9 $$\begin{aligned} v_{ij}(M_\mathrm{inv})&=-C_{ij}\frac{1}{4f^2}(2M_\mathrm{inv}-m_i-m_j) \nonumber \\&\quad \times \left( \frac{m_i+E_i}{2m_i}\right) ^{1/2} \left( \frac{m_j+E_j}{2m_j}\right) ^{1/2}, \end{aligned}$$

where $f$ is the averaged meson decay constant $f = 1.123f_\pi$ [38] with $f_\pi = 92.4$ MeV, $E_i$ ($m_i$) the energies (masses) of the baryons of the $i$th channel and the $C_{ij}$ are coefficients, which for isospin $I=0$ are given by

10 $$\begin{aligned} C_{ij} =\begin{pmatrix} 3 &{} -\sqrt{\frac{3}{2}} \\ -\sqrt{\frac{3}{2}}&{} 4 \end{pmatrix} , \end{aligned}$$

where the $i$ and $j$ subscripts stand for $\bar{K} N$ and $\pi \Sigma$ in an isospin basis. Equations (9) and (10) represent the standard Weinberg–Tomozawa interaction, slightly modified to incorporate relativistic corrections [38]. Note that we work in an isospin-symmetric formalism for the meson–baryon interaction. Further, the values of the elements of the matrix $C_{ij}$ are given by chiral symmetry. The other meson–baryon channels in $I=0$ and strangeness $S=-1$, $\eta \Lambda$ and $K\Xi$, are not explicitly included. Indeed, since the thresholds of these channels lay far above from the energies that we will consider in the present work, they can effectively be reabsorbed in the regularization parameters that we will explain below.

In Refs. [63, 64] the coefficient matrix $C_{ij}$, Eq. (10), was substituted by

11 $$\begin{aligned} C_{ij} = \begin{pmatrix} 3 \alpha _{11} &{} -\sqrt{\frac{3}{2}} \alpha _{12} \\ -\sqrt{\frac{3}{2}}\alpha _{12}&{} 4 \alpha _{22} \end{pmatrix}, \end{aligned}$$

where the parameters $\alpha _i$ were to be fitted to $\gamma p \rightarrow K^+ \pi \Sigma$ experimental data. In this way one is allowed to fine tune the theoretical chiral unitary inspired model, incorporating in an effective way possible contributions of higher order terms, and extract from experiment an accurate position for the two $\Lambda (1405)$ poles and the actual shape of the meson–baryon scattering amplitudes.

On the other hand, the $G_i$ function in Eq. (8) [as defined in Eq. (6)] can be regularized either with a three-momentum cutoff or with dimensional regularization in terms of subtraction constants, $a_i$, one for each meson–baryon channel. In Refs. [63, 64] these parameters were also allowed to vary slightly substituting them by $a_{KN}\rightarrow \beta _1 a_{KN}$, $a_{\pi \Sigma }\rightarrow \beta _2 a_{\pi \Sigma }$ with $a_{KN} = -1.84$, $a_{\pi \Sigma }=-2$ [38, 40]. All in all, there are only five $\alpha _i$, $\beta _i$, parameters needed in the present work. Their values are taken from Table I in [64].

When looking for poles in the second Riemann sheet of the complex energy plane, the amplitudes of this model provide the $\Lambda (1405)$ pole positions at $1352-48i$ and $1419-29i$ MeV. Note that the parameters do not differ much from one, as would be expected if reality is not far from the predictions of the chiral unitary theory. In the $\bar{K} N\rightarrow \bar{K} N$ amplitude the highest pole is more pronounced. The $\pi \Sigma \rightarrow \pi \Sigma$ amplitude picks more the lowest pole while in the $\bar{K} N\rightarrow \pi \Sigma$ amplitude a more balanced mixture between both poles is visible but with a larger weight of the highest one. (See, for instance, Fig. 6 in Ref. [64].) All this is reminiscent of the fact that the highest pole couples dominantly to $\bar{K} N$ and the lowest pole to $\pi \Sigma$, see Table II in Ref. [64].

## Results

**Fig. 3:**
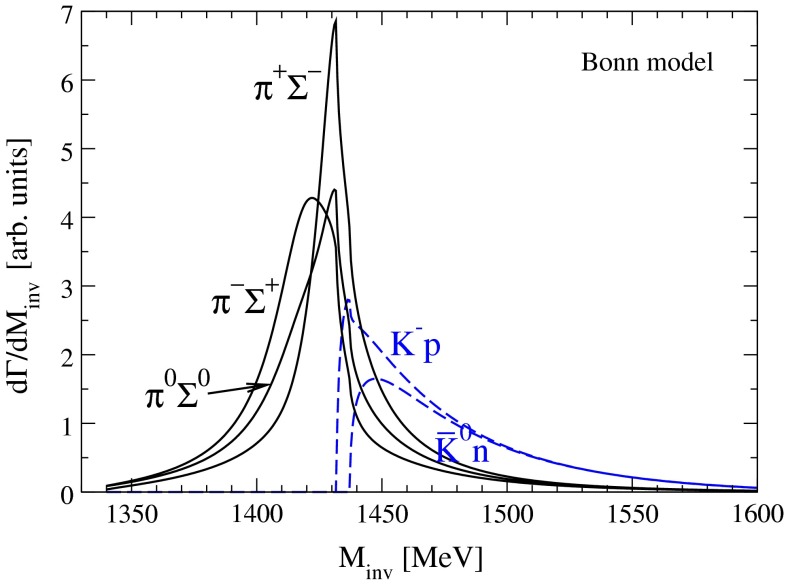
Invariant mass distribution within the Bonn model considering isospin breaking

After having set up the framework, we present here our predictions for the $\pi \Sigma$ and $\bar{K} N$ invariant mass distributions from the $\Lambda _b$ decay. As mentioned before, one of the important features^3^ of the present study is quantification of the theoretical uncertainties, due to different meson–baryon models. To make this comparison more meaningful, the trivial sources of differences must be studied first, such as isospin symmetry. The latter is implemented in the MV model by construction, while it is broken explicitly in the Bonn model. The isospin breaking in the Bonn model arises naturally due to chiral potential of the next-to-leading order. All particle masses are considered to be the physical ones; see the discussion in Refs. [47, 65, 75]. In Fig. 3 we show the results for the Bonn model considering the explicit isospin breaking. This provides the order of the correction for the subsequent figures if one considers isospin breaking. In the following we will consider the isospin-symmetric case for simplicity and to ease the comparison with the MV model.

**Fig. 4:**
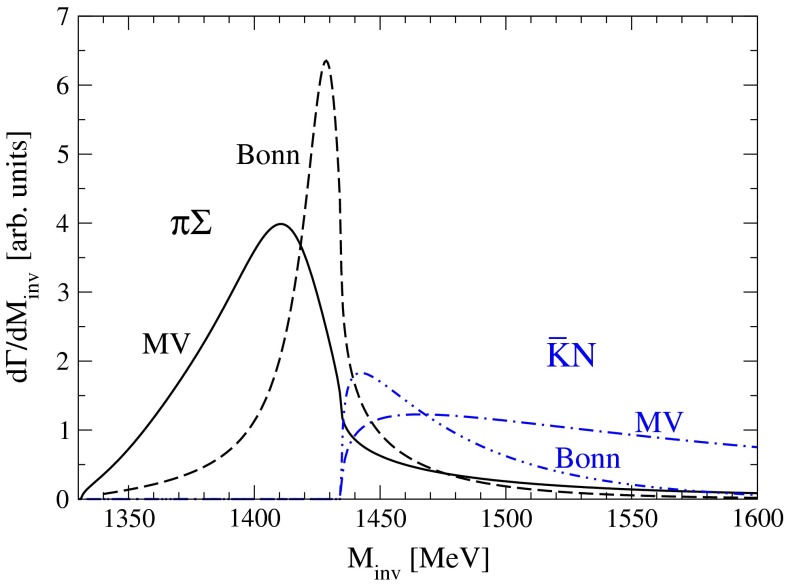
Results for the $\pi \Sigma$ and $\bar{K}N$ invariant mass distributions for the $\Lambda _b \rightarrow J/\psi \,\pi \Sigma$ and $\Lambda _b \rightarrow J/\psi \,\bar{K}N$ decays, respectively, for both models considered in the present work

In Fig. 4 we show the final results for both the Bonn and the MV models. In the $\pi \Sigma$ final-state channel the peak of the $\Lambda (1405)$ is clearly visible. In fact, this is mostly due to the higher mass pole of the $\Lambda (1405)$ since the contribution proportional to $t_{\bar{K} N,\pi \Sigma }$ of Eq. (2) is the dominant one. The difference in the $\pi \Sigma$ mass distribution between both models is reminiscent of the fact that, as explained above, the Bonn model gets a narrower ($24\text { MeV}$) highest $\Lambda (1405)$ pole than the MV model ($58\text { MeV}$).

In the $\bar{K} N$ final state, the dominant contribution comes from the part proportional to $t_{\bar{K} N,\bar{K} N}$ which again is more sensitive to the higher mass $\Lambda (1405)$ pole. However, in this channel only the effect of the tail of the resonance is visible since the threshold of the $\bar{K} N$ mass distribution is located above the position of the $\Lambda (1405)$ peak. Nevertheless, that tail is enough to provide a high strength close to the threshold, what makes the line shape of the $\bar{K} N$ invariant mass distribution to be very different from just a phase-space distribution. The dependence on the choice of the model in this channel is due to the fact that the highest pole is slightly closer to threshold in the Bonn model compared to the MV one. Because of this feature, the Bonn model produces a narrower bump close to $\bar{K} N$ invariant mass threshold than the MV one. This observable is then very sensitive to the exact position of the resonance pole, due to the proximity between the threshold and the pole. As mentioned in the introduction, different reactions can reflect different weights for both poles of the $\Lambda (1405)$ resonance, depending on the particular production dynamics. In the present case, the highest pole is the one that shows up dominantly.

On the other hand, the qualitative agreement in Fig. 4 of the results between the MV and Bonn models is quite remarkable, given their theoretical differences and fitting strategies as explained before. Nonetheless we can regard the difference between the models as the main source of the theoretical uncertainty.

While the overall normalization of the invariant mass distributions is unknown, the shape and the ratio between the $\pi \Sigma$ and $\bar{K} N$ distributions is unchanged and it is a genuine prediction of the present work. Indeed, the ratio between the maximum values of the $\pi \Sigma$ and $\bar{K} N$ distribution is 3.3 for the MV and 3.5 for the Bonn model. The value of that ratio as well as the shape of the distributions are then genuine predictions of the chiral unitary approach. As already stated, the differences between the different curves can be considered as an estimation of the theoretical uncertainty. In conclusion, Fig. 4 serves to predict the invariant mass distributions of either $\pi \Sigma$ or $\bar{K} N$, once the absolute normalization of the mass distribution of the other channel has been measured. For instance, if the LHCb [27] and CDF [29] collaboration were to measure the $K^-p$ mass distribution in the $\Lambda _b \rightarrow J/\psi ~ K^- p$ decay, then the shape should agree with the prediction of Fig. 4 and once normalized, the $\bar{K} N$ and $\pi \Sigma$ distributions would be given both in size and shape.

## Summary

We have carried out a theoretical study of the $\Lambda (1405)$ production in the $\Lambda _b \rightarrow J/\psi \,\pi \Sigma$ and $\Lambda _b \rightarrow J/\psi \,\bar{K} N$ decays. The initial weak production at the level of quarks to give a $c\bar{c}$ for the $J/\Psi$ and three quarks $uds$ is the same for both channels and then irrelevant in the relative ratio. The hadronization of the $uds$ into the different meson–baryon channels is then implemented and the different channels are related using suitable $\mathrm{SU}(3)$ arguments.

The key point of the chiral unitary models is that the $\Lambda (1405)$ comes out as dynamically generated. Actually, two poles are predicted for this resonance. Accordingly, we implement the final-state interaction of the meson–baryon pair, using two different theoretical models [64, 65]. The MV model [63, 64] uses as the kernel of the unitarization procedure the lowest order meson–baryon chiral Lagrangian slightly modified to fit photoproduction data. On the other hand, the Bonn model [65] includes in the kernel from higher order meson–baryon Lagrangians fitted to photoproduction and meson–baryon cross section data.

The $\Lambda (1405)$ resonant shape is clearly visible in the $\pi \Sigma$ mass distribution and its tail distorts considerably the $\bar{K} N$ distribution in spite of the pole being below the $\bar{K} N$ threshold. This particular decay is mostly influenced by the higher mass pole of the $\Lambda (1405)$ resonance. Therefore this decay is specially suited to study the properties of the high mass $\Lambda (1405)$ resonance both theoretically and experimentally.

The results for both theoretical models used in the present work are qualitatively similar and their differences can be considered as the theoretical uncertainty of this calculation. The line shapes of the $\pi \Sigma$ and $\bar{K} N$ distributions and their relative strengths are predictions of this model which could be compared to future experimental measurements amenable to study the $\Lambda (1405)$ resonance in this decay.

## Footnotes

1 In fact, one might thus speak of two $\Lambda (1405)$ particles.

2 More precisely, $t_{ij}$ denotes the s-wave contribution to the scattering matrix.

3 Which is unfortunately not very common in such studies.
